# Increasing Cyetpyrafen Spray Volume and Ozone Spray Improves the Control Effects against Two-Spotted Spider Mite (*Tetranychus urticae*) in Strawberries

**DOI:** 10.3390/plants13131792

**Published:** 2024-06-28

**Authors:** Lili Jiang, Hairong Wang, Kang Qiao, Chong Wu

**Affiliations:** 1 Shandong Institute of Pomology, Tai’an 271000, China; gssjianglili@shandong.cn (L.J.); whr_sdip@163.com (H.W.); 2 Key Laboratory of Pesticide Toxicology & Application Technique, College of Plant Protection, Shandong Agricultural University, Tai’an 271018, China

**Keywords:** *Tetranychus urticae*, cyenopyrafen, ozone spray

## Abstract

The two-spotted spider mite (*Tetranychus urticae*) is a constant threat to greenhouse strawberry production. The application of synthetic acaricides is the main method of controlling *T. urticae*. However, resistance development to traditional acaricides reduces their efficacy and eventually leads to control failure. It is important for strawberry growers to look for new acaricides and application technologies that can limit the harmfulness of *T. urticae* in environmentally friendly ways. In the current study, laboratory toxicity tests and field trials were performed to screen high-efficiency acaricides, and then application technologies were improved to enhance the management of *T. urticae*. In the laboratory toxicity tests, the results showed that the LC_50_ (median lethal concentration) value of cyetpyrafen, cyenopyrafen, cyflumetofen, bifenazate, abamectin, azocyclotin, pyridaben, spirodiclofen, and etoxazole against adult *T. urticae* was 0.226, 0.240, 0.415, 3.583, 5.531, 25.58, 39.69, 140.3, and 267.7 mg/L, respectively. In addition, the LC_50_ value of the nine acaricides against eggs of *T. urticae* was 0.082, 0.097, 0.931, 18.56, 25.52, 45.61, 36.32, 1.954, and 0.040 mg/L, respectively. The field trial results showed that the best control effect was obtained in cyetpyrafen at 300 mL/ha treatment. Cyetpyrafen was chosen for further application technology tests. In the spray volume tests, the results showed that increasing the spray volume from 900 to 1050 L/ha significantly improved the control of *T. urticae*. In addition, the results from the spray instrument tests demonstrated that the control effects on *T. urticae* in the ozone spray treatments were significantly higher than those of the conventional and electrostatic sprays 1 and 3 days after treatment (DAT). Therefore, this study suggested that cyetpyrafen effectively controlled *T. urticae* both in the laboratory tests and in the field trials. Increasing the spray volume and application of ozone spray significantly improved *T. urticae* management.

## 1. Introduction

The strawberry (*Fragaria* × *ananassa*) is the most economically important soft fruit species in the world. As the largest strawberry producer and consumer, over 3,981,600 t of strawberry were harvested on 147,450 ha in China, with a gross output value of over 60 billion CNY in 2022 [1]. Strawberry production has had a 5–7% annual increase in China over the past six years. However, with the development of the strawberry industry, pests have become the main problem. Particularly, the two-spotted spider mite (*Tetranychus urticae*) is considered a constant threat to sustainable strawberry production [2,3]. *T. urticae* is a polyphagous pest with a short life cycle, a high fecundity, and a great ability to develop acaricide resistance [4,5,6]. The host range of *T. urticae* covers over 1400 plant species from more than 140 different plant families [7]. *T. urticae* pests feed mainly on the underside of leaves, leading to reduced photosynthesis and increased transpiration [8]. Due to feeding damage, strawberry quality and quantity can be seriously reduced, with a typical yield loss of 50–80% [9].

Resistant cultivars are the first choice in managing *T. urticae* [10]. However, the most popular strawberry cultivars in local markets vary significantly in their sensitivity to *T. urticae* [11]. Moreover, cultural and biocontrol practices are promising strategies, but they have a stability problem under field conditions [12,13]. Commonly, the management of *T. urticae* relies mainly on the application of chemical pesticides or acaricides due to their easy application and low economic costs. However, this method is not always effective, as *T. urticae* has a rapid development of resistance to chemical products [4]. Currently, *T. urticae* develops resistance to most selective acaricides, including more than 96 active ingredients with different modes of action [5,14,15]. Thus, the search for environmentally friendly and highly efficient acaricides is urgently needed for growers to control *T. urticae* in strawberry production [13].

Many new acaricides have recently been developed for their acaricidal effect in *T. urticae* management. Cyetpyrafen is the latest acaricide with a mode of action as a complex II (succinate-ubiquinone oxidoreductase) inhibitor in the respiratory chain, firstly commercialized in China in 2017 [16]. It was obtained using cyenopyrafen as the lead compound, by structural modification of the pyrazole ring and hydroxyl portion, having high acaricide activity against all developmental stages of *T. urticae*. In a recent study, the uptake and metabolism of cyetpyrafen in plants were explored, and the results showed that 13 transformation products of cyetpyrafen had been identified [17]. Cyenopyrafen is a beta-ketonitrile derivative and a recently developed acaricide with the same mode of action as cyetpyrafen [18]. Cyflumetofen is a benzoyl acetonitrile acaricide circulated by Otsuka AgriTechno Co., Ltd. [19]. The above-mentioned acaricides have great potential to control *T. urticae*. On the other hand, pesticide application technologies, including the ozone and electrostatic spray methods, have developed rapidly in recent years [20,21]. Diversifying control methods is a pivotal strategy in effectively coping with acaricide resistance. In the current study, laboratory tests and field trials were used to screen highly efficacious acaricides to manage *T. urticae*. Then, the application technologies were optimized to improve their control efficacy on *T. urticae*. Our findings are valuable for growers optimizing management strategies to control *T. urticae* sustainably in field conditions.

## 2. Results

### 2.1. Laboratory Toxicity Tests

The laboratory toxicity results showed that the LC_50_ value of cyetpyrafen, cyenopyrafen, cyflumetofen, bifenazate, abamectin, azocyclotin, pyridaben, spirodiclofen, and etoxazole against adult *T. urticae* was 0.226, 0.240, 0.415, 3.583, 5.531, 25.58, 39.69, 140.3, and 267.7 mg/L, respectively (Table 1). In addition, the LC_50_ value of the nine acaricides to the eggs of *T. urticae* was 0.082, 0.097, 0.931, 18.56, 25.52, 45.61, 36.32, 1.954, and 0.040 mg/L, respectively. After an integrated analysis, we further conducted field trials to confirm the control effects of five acaricides, including cyetpyrafen, cyenopyrafen, cyflumetofen, bifenazate, and etoxazole.

### 2.2. Field Trials

**Figure 1:**
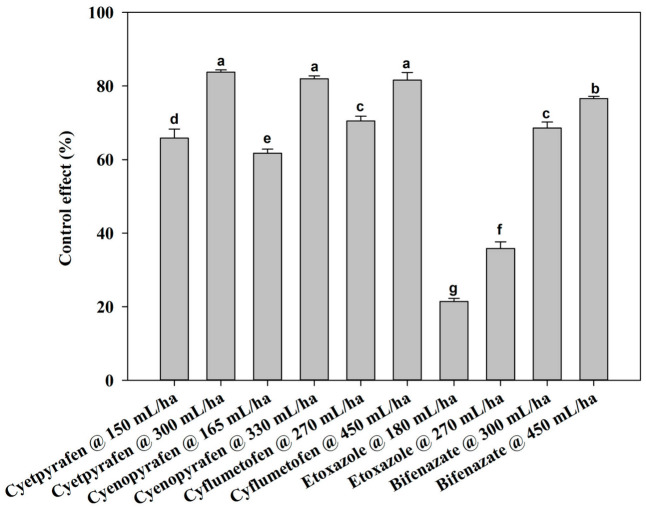
Control effect of acaricides on *Tetranychus urticae* in field trials one day after treatment. Control effect (%) = (the decrease rate in the control mite population − the decrease rate in the treated mite population)/(100 − the decrease rate in the control mite population) × 100. The data are represented as the mean ± standard deviation of the mean and separated with Tukey’s HSD test. The same letters above the columns indicate that the values are not statistically different (*p* < 0.05).

**Figure 2:**
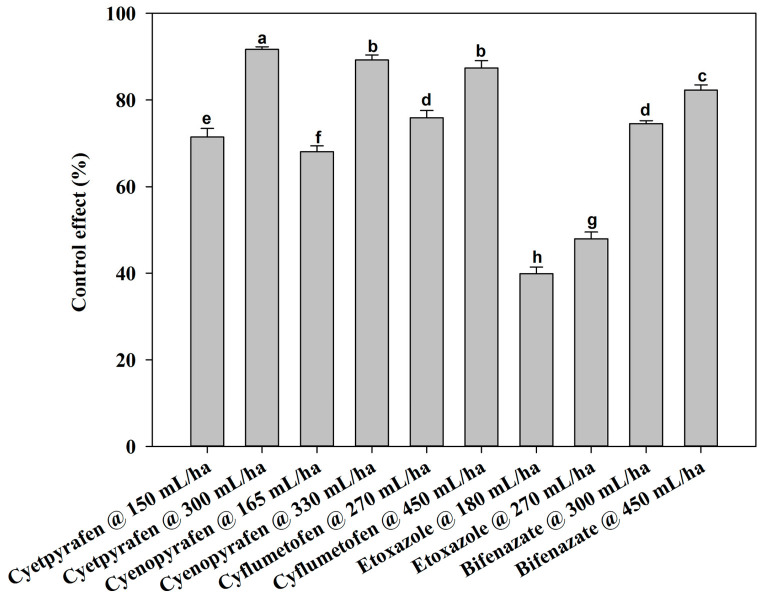
Control effect of acaricides on *Tetranychus urticae* in field trials three days after treatment. Control effect (%) = (the decrease rate in the control mite population − the decrease rate in the treated mite population)/(100 − the decrease rate in the control mite population) × 100. The data are represented as the mean ± standard deviation of the mean and separated with Tukey’s HSD test. The same letters above the columns indicate that the values are not statistically different (*p* < 0.05).

**Figure 3:**
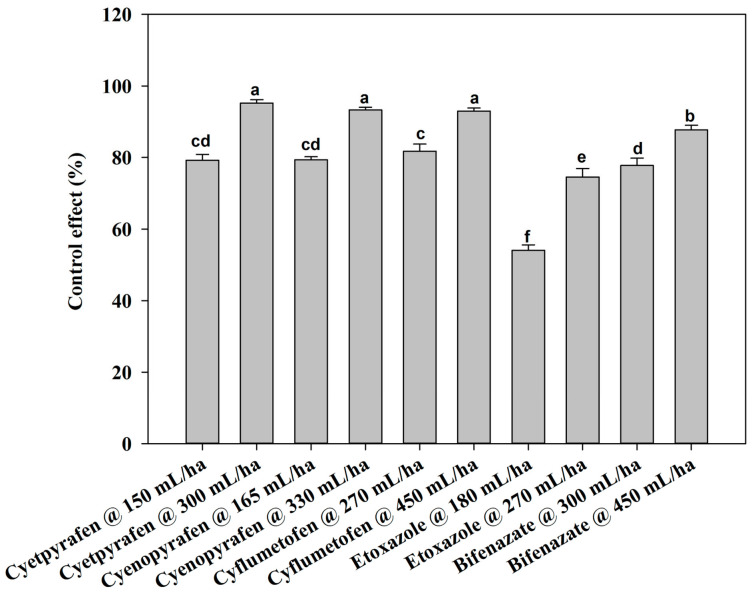
Control effect of acaricides on *Tetranychus urticae* in field trials seven days after treatment. Control effect (%) = (the decrease rate in the control mite population− the decrease rate in the treated mite population)/(100 − the decrease rate in the control mite population) × 100. The data are represented as the mean ± standard deviation of the mean and separated with Tukey’s HSD test. The same letters above the columns indicate that the values are not statistically different (*p* < 0.05).

**Figure 4:**
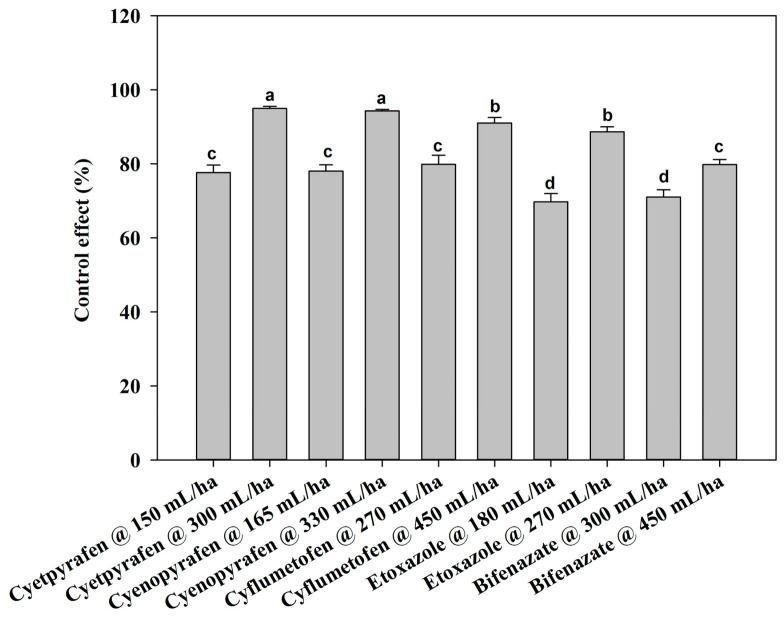
Control effect of acaricides on *Tetranychus urticae* in field trials fourteen days after treatment. Control effect (%) = (the decrease rate in the control mite population − the decrease rate in the treated mite population)/(100 − the decrease rate in the control mite population) × 100. The data are represented as the mean ± standard deviation of the mean and separated with Tukey’s HSD test. The same letters above the columns indicate that the values are not statistically different (*p* < 0.05).

There were no notable differences between the two-year field trials, so the data were pooled. At one day after treatment (DAT), cyetpyrafen at 300 mL/ha, cyenopyrafen at 330 mL/ha, and cyflumetofen at 450 g/ha were most effective in decreasing the mite population, with a control of 83.77%, 81.99%, and 81.57%, respectively, which was significantly better compared to the other treatments (Figure 1). Moreover, the control effect of etoxazole at 180 mL/ha was inferior to that of the other treatments. The remaining treatments had medium effects. At 3 DAT, cyetpyrafen at 300 mL/ha had the best control effect (Figure 2). Cyenopyrafen at 330 mL/ha and cyflumetofen at 450 g/ha had slightly weaker control effects in reducing the population of mites, but they were better than bifenazate at 450 mL/ha and the other treatments. At 7 and 14 DAT, a similar trend to 1 DAT was observed (Figure 3 and Figure 4). However, etoxazole at 180 mL/ha had the lowest control effects. Overall, the higher rates of cyetpyrafen, cyenopyrafen, and cyflumetofen had better control effects and lasted longer compared to the other treatments.

### 2.3. Application Technology Tests

**Figure 5:**
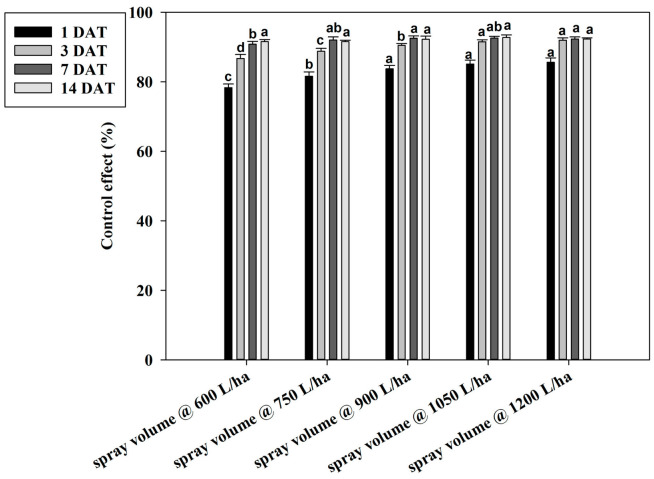
Control effect of different spray volumes on *Tetranychus urticae* in field trials. Control effect (%) = (the decrease rate in the control mite population − the decrease rate in the treated mite population)/(100 − the decrease rate in the control mite population) × 100. The data are represented as the mean ± standard deviation of the mean and separated with Tukey’s HSD test. The same letters above the columns indicate that the values are not statistically different (*p* < 0.05).

Based on the above results, the best treatment—cyetpyrafen at 300 mL/ha—was used in the field trials to verify the better spray volume and method for improving the management of *T. urticae*. At 1 and 3 DAT, the control effects on *T. urticae* in strawberries receiving spray volumes of 600 and 750 L/ha were 78.35% and 82.59% and 81.60% and 84.40%, respectively, which were significantly lower values than those of a conventional spray with a volume of 900 L/ha (83.01% and 85.46%, respectively) (Figure 5). However, the control effects on *T. urticae* at the spray volumes of 1050 and 1200 L/ha were notably (*p* < 0.05) higher than those of a conventional spray. At 7 DAT, the control effect of the spray volume of 600 L/ha was 90.80%, which was lower than that of a volume of 900 L/ha. There were no differences between the other treatments. At 14 DAT, no significantly differences were observed among all the treatments.

**Figure 6:**
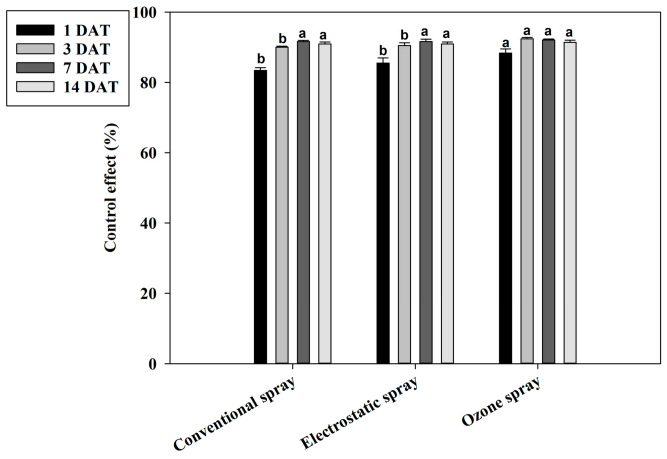
Control effect of the spray instrument on *Tetranychus urticae* in field trials. Control effect (%) = (the decrease rate in the control mite population − the decrease rate in the treated mite population)/(100 − the decrease rate in the control mite population) × 100. The data are represented as the mean ± standard deviation of the mean and separated with Tukey’s HSD test. The same letters above the columns indicate that the values are not statistically different (*p* < 0.05).

The control effects on *T. urticae* were further analyzed for different spray methods. The results showed that the control effects on *T. urticae* in the ozone spray treatments (88.35% and 92.41%, respectively) were significantly higher compared to the conventional spray (83.43% and 90.01%, respectively) and the electrostatic spray (85.54% and 90.51%, respectively) at 1 and 3 DAT (Figure 6). However, these differences disappeared at 7 and 14 DAT.

## 3. Discussion

*T. urticae* is a serious threat to the sustainable production of strawberries globally. Control of *T. urticae* is heavily reliant on chemical acaricides. However, *T. urticae* is a hard-to-control pest due to its rapid acaricide resistance [22]. The success of *T. urticae* in agroecosystems also benefits from its very broad plant host range, extremely short generation cycle, strong reproductive ability, arrhenotokous parthenogenesis, and overwintering strategy [14]. As climate change continues to cause higher temperatures and drier conditions worldwide, *T. urticae* control will become increasingly challenging. Therefore, it is critical to explore new acaricides with ovicidal and adulticidal effects and novel pesticide application strategies to manage *T. urticae*.

In the present study, in vitro toxicity tests against *T. urticae* were first performed to screen for highly efficient acaricides to control *T. urticae*. The results indicated that cyetpyrafen, cyenopyrafen, and cyflumetofen had strong lethal effects against *T. urticae* adults. In addition, etoxazole, cyetpyrafen, cyenopyrafen, and cyflumetofen exhibited high ovicidal activity against *T. urticae*.

Cyetpyrafen is the newest product among complex II class mitochondrial electron transport inhibitors [16]. It is a derivative synthesized from the lead compound cyenopyrafen, and it exhibits a more effective acaricidal activity than cyenopyrafen [16]. Cyflumetofen is a novel acaricide with the same mode of action as cyetpyrafen [19]. Bifenazate is a hydrazine carbazate acaricide discovered in 1990, acting via the inhibition of complex III [23]. Meanwhile, etoxazole, a member of the diphenyl oxazoline class of acaricides, belongs to those acaricides that are only toxic to the eggs, larvae, and nymphs of *T. urticae*, but it lacks efficacy against male and female adults [24]. All of the above are the most frequently used acaricides in China. However, the paradox is that most of the acaricides available are only effective for either the eggs or adults of *T. urticae*. Given the importance of knowledge about *T. urticae* control, there is an urgent need for screening new active acaricides with both ovicidal and adulticidal effects. Cyetpyrafen has high acaricide activity against all developmental stages of this mite, which shows great advantages.

To verify the control effect of these treatments, field trials were performed to confirm the control performance of the five selected acaricides against *T. urticae*. The results showed that cyetpyrafen had a higher efficacy than the other acaricides at different sampling times. These results agreed with Sun et al. [25], who reported that cyetpyrafen was one of the most widely used acaricides for *T. urticae* control in China at the time of writing. Moreover, its high safety for non-target organisms makes it suitable for integrated pest management [26]. Ouyang et al. [26] reported that the application of cyetpyrafen had no effects on the population growth of the predatory mite *Neoseiulus californicus*. Therefore, cyetpyrafen was reasonably favored by farmers and used frequently and widely on agricultural crops. However, Chen et al. [27] reported that a low level of resistance of *T. urticae* against cyetpyrafen was detected in China. This was further verified by Sun et al. [25], who reported that cyetpyrafen-resistant *T. urticae* showed serious cross-resistance to cyenopyrafen and cyflumetofen. In addition, Ding et al. [28] reported that *Panonychus citri* (McGregor) had developed significant resistance to cyetpyrafen and that the overexpression of *PcGSTO1* was associated with cyetpyrafen resistance in *P. citri*. Thus, the reasonable application of cyetpyrafen alongside other strategies and resistance monitoring should be carried out to extend the product lifetime of cyetpyrafen [29]. The availability of compounds with different modes of action that can be used in alternation is of crucial importance for managing acaricide resistance. Combining cyetpyrafen with other selective acaricides such as spirodiclofen is a potential method of controlling *T. urticae*. This work needs further validation.

Based on our results, cyetpyrafen demonstrated the effective management of *T. urticae* in both the in vitro tests and in the field trials. Therefore, cyetpyrafen was further investigated in the application technology tests. The results from the spray volume study indicated that, at 1 and 3 DAT, spray volumes of 1050 and 1200 L/ha notably improved the management of *T. urticae* compared to the conventional spray volume (900 L/ha) when the rate of cyetpyrafen remained unchanged. This may have been due to the fact that most of the acaricides available are not systemic, so a higher spray volume means a higher distribution of acaricides, leading to higher control effects [13]. In addition, a higher spray volume increases the humidity, which is harmful to *T. urticae*.

Many studies have indicated that a spray instrument such as an electrostatic spray improves the uniformity of pesticide distribution on plants [30,31]. The results from this study demonstrated no notable differences between the conventional spray and the electrostatic spray. As we all know, ozone spray has many advantages over a conventional spray [32,33]. Ozone spray is considered an environmentally friendly and effective alternative to the conventional spray. Because of the short half-life and easy conversion to oxygen of ozone spray, there are no toxic compounds on the treated products. As an environmentally friendly approach, ozone spray is suitable for use in fresh and soft fruits, such as strawberries. This result was partly in agreement with our previous findings, whereby the index of strawberry powdery mildew in ozone spray was notably reduced compared to conventional and electrostatic sprays [34].

It is worth noting that the discovery rate and commercialization of new acaricides are hindered due to regulatory and economic hurdles. One of the sustainable, long-term perspectives for acaricide application is a mixture of acaricides with different modes of action, interacting with other control tactics. Besides the implementation of advanced technologies for acaricide application, a wider use of microbial and phytogenic products with acaricidal activity could contribute to a more effective *T. urticae* control in the future. Tanaka et al. [35] demonstrated that combined night-time ultraviolet B irradiation and phytoseiid mite application offered optimal control of *T. urticae*.

Overall, in this study, we concluded that cyetpyrafen could control *T. urticae* effectively. The integrated application of cyetpyrafen with ozone spray and an appropriately increased spray volume improved the control effect on *T. urticae*. Further studies need to be conducted to explore the potential of cyetpyrafen in combination with other selective acaricides in the management of *T. urticae*.

## 4. Materials and Methods

### 4.1. Chemicals

The technical materials and formulations of cyetpyrafen, cyenopyrafen, bifenazate, abamectin, azocyclotin, spirodiclofen, cyflumetofen, and pyridaben were purchased from different companies (Table 2).

### 4.2. Testing Plant Cultivation and Mite Strain

Strawberries cv. Akihime in greenhouses at the Jinniushan Experimental Base of the Shandong Institute of Pomology (36°05′53.31″ N, 116°57′53.16″ E) were used to rear mites in a growth chamber at 24 ± 2 °C, a relative humidity (RH) of 72 ± 3%, and a photoperiod of L16:D8 h. The population of *T. urticae* was originally collected in 2018 and maintained on fresh strawberry leaves in the above-mentioned growth chamber without any further acaricide exposure.

### 4.3. Laboratory Toxicity Tests

The leaf-disc spray method was used to evaluate the acaricidal activity using different developmental stages of *T. urticae* [36]. All the technical materials of the acaricides listed in Table 1 were dissolved in acetone to obtain stock solutions at 1.0 × 10^4^ mg/L and stored at 6 °C before use. A series of doses (0.5, 1.0, 5.0, 10, 25, and 50 mg a.i./L) of the acaricides were prepared in solutions of acetone and 0.1% Tween 80 (*v*/*v*).

#### 4.3.1. Adults of *T. urticae*

Intact leaves of strawberries cv. Akihime without any exposure to acaricides were transferred into 9 cm Petri dishes, with the upper surfaces of the leaves placed downwards on water-saturated cotton. Forty adults of *T. urticae* were transferred onto the strawberry leaves. After 30 min, the survival of *T. urticae* specimens was observed under an Olympus SZX10 stereoscopic microscope (Tokyo, Japan). Twenty adult mites per leaf remained, and the acaricides were applied by spraying in a 3WPSH-500E Potter-type tower (Nanjing Institute of Agricultural Mechanization, Nanjing, China). One mL of acaricides at different concentrations was sprayed on a Petri dish with sterile distilled water, and a mixture of water with 0.1% Tween 80 was served as the untreated control. The number of immobile mites was recorded using an Olympus SZX10 stereoscopic microscope after 24 h. All the experiments were repeated thrice using a randomized complete block design, and each treatment consisted of four replicates. The mortality rate was calculated using the following formula: mite population decrease rate (%) = (number of *T. urticae* before treatment − number of *T. urticae* after treatment/number of *T. urticae* before treatment) × 100. The mortality in the control group was expected to be less than 20%.

#### 4.3.2. Eggs of *T. urticae*

Intact leaves of strawberries cv. Akihime without any exposure to acaricides were picked and cleaned with sterile distilled water. Then, the leaves were transferred into 9 cm Petri dishes, with their upper surfaces placed downwards on water-saturated cotton, and placed in the dark in a laboratory incubator at 25 ± 1 °C and an RH of 65 ± 5%. Forty adult female mites were transferred onto the leaves and allowed to oviposit for 24 h, and then they were removed from the leaves. One mL of acaricides at different concentrations was sprayed on each Petri dish with sterile distilled water, and a mixture of water with 0.1% Tween 80 was served as the untreated control, using a Potter-type tower. After treatment, the number of eggs was recorded using an Olympus SZX10 stereoscopic microscope. Fifty eggs were kept on each leaf, and the others were removed. The number of hatched eggs was counted every day until the hatchability exceeded 85%. The hatchability was calculated using the following formula: Hatchability (%) = number of hatched eggs/50 × 100. Inhibition rate of hatchability (%) = (hatchability of untreated control − hatchability of treatment)/hatchability of untreated control × 100. All the experiments were repeated thrice using a randomized complete block design, and each treatment consisted of four replicates.

### 4.4. Field Trials

Field trials were conducted in 2021 and 2022 at the Jinniushan Experimental Base. The field trials were conducted in the winter-warm greenhouse with a temperature of 20 ± 2 °C and an RH of 65% during the day and a temperature of 10 ± 2 °C and an RH of 80% at night. On September 5, in 2021, and on September 8, in 2022, three true-leaf strawberry (Akihime) plants were transplanted into a 0.4 m depth planter containing a mixture of perlite and vermiculite, with an in-row spacing of 0.15 m, under high plastic tunnels. There were 60 planters per 667 m^2^, with 100 strawberries in each planter.

All eleven treatments were arranged in a randomized complete block design, with three replicates: (1–2) 30% cyetpyrafen SC applied at 150 and 300 mL/ha; (3–4) 30% cyenopyrafen SC applied at 165 and 330 mL/ha; (5–6) 20% cyflumetofen SC applied at 270 and 450 mL/ha; (7–8) 43% bifenazate SC applied at 300 and 450 mL/ha; (9–10) 30% etoxazole SC applied at 180 and 270 mL/ha; and (11) an untreated control (CK).

On 15 November 2021, and on 12 November 2022, all the treatments were sprayed with a 3WBJ-16DZ electrostatic sprayer mounted with a hollow cone nozzle (JiaLe, Suzhou, China) at the conventional spray volume (900 L/ha) under an operating pressure of 0.5 Mpa. Conventional cultivation and management was employed, and the overshadow was applied at noon to slow seedling. Commercial strawberry production standards, including irrigation, plant protection, and fertilization practices, were used [31].

Five sites were selected in each plot, and five leaves were selected in each site. The number of adults of *T. urticae* was recorded before the treatments. One, three, seven, and fourteen days after the treatments (DATs), the number was rated, and the control effect was determined. The decrease rate in the mite population was calculated as follows: Control effect (%) = (the decrease rate in the control mite population − the decrease rate in the treated mite population)/(100 − the decrease rate in the control mite population) × 100. The decrease rate = (the number of mites before treatment − the number of mites after treatment)/the number of mites before treatment × 100.

### 4.5. Application Technology Tests

Based on the above results, application technology tests, including spray volume and spray method tests, were conducted for the acaricide cyetpyrafen from March to May, in 2022 and 2023, at the Jinniushan Experimental Base. In the spray volume tests, the control effect of different spray volumes was compared in the context of the same acaricide application rate (cyetpyrafen at 300 mL/ha, the most effective treatment under field conditions). The experiments were laid in a randomized complete block design with three replicates. The decrease rate in the mite population was calculated as described above.

The effects of different spray methods, including conventional, electrostatic, and ozone sprays, were determined. The conventional and electrostatic spray methods were tested with a 3WBJ-16DZ electrostatic sprayer (JiaLe, Suzhou, China). The ozone spray was applied with a MH-O3-1 ozone sprayer (Zhejiang Menghua, Taizhou, China). The spray volume was 900 L/ha. The experiments were laid in a randomized complete block design with three replicates. The decrease rate in the mite population was calculated as described earlier.

### 4.6. Statistical Analysis

All statistical analyses were performed using SPSS (V17, IBM SPSS Statistics, Chicago, IL, USA). The LC_50_ (median lethal concentration) values were obtained by a probit analysis in SPSS with a Log_10_ transformation of the acaricide doses. For the non-parametric data, a Kruskal–Wallis H-test was performed. The data collected from the laboratory experiments and the field trials were analyzed for the homogeneity of variances, and the data were pooled after the tests for equal variances. The data were subjected to a one-way analysis of variance (ANOVA). The differences between the treatment means were examined using Tukey’s HSD test (*p* < 0.05).

## Figures and Tables

**Table 1:** Toxicity of nine acaricides to adults and eggs of *Tetranychus urticae*.

Acaricides	*T. urticae*	Regression (Y=)	*R* ^2^	LC_50_ (mg/L) *^a^*
Cyetpyrafen	adults	1.449x + 0.936	0.912	0.226
	eggs	1.342x + 1.459	0.979	0.082
Cyenopyrafen	adults	1.385x + 0.858	0.975	0.240
	eggs	1.526x + 1.544	0.840	0.097
Cyflumetofen	adults	1.648x + 0.630	0.957	0.415
	eggs	1.769x + 1.437	0.154	0.931
Bifenazate	adults	1.583x − 0.877	0.869	3.583
	eggs	1.604x − 2.035	0.949	18.56
Abamectin	adults	1.750x − 1.300	0.986	5.531
	eggs	1.639x − 2.306	0.962	25.52
Azocyclotin	adults	1.541x − 2.244	0.964	25.58
	eggs	1.531x − 2.540	0.937	45.61
Pyridaben	adults	1.835x − 2.934	0.957	39.69
	eggs	1.542x − 2.405	0.908	36.32
Spirodiclofen	adults	1.388x − 2.980	0.975	140.3
	eggs	1.681x − 0.489	0.901	1.954
Etoxazole	adults	1.755x − 4.260	0.913	267.7
	eggs	1.434x + 2.001	0.952	0.040

*^a^* The LC_50_ values have been calculated by a probit analysis in SPSS using the inhibition rate against the Log_10_ value of the acaricide concentrations.

**Table 2:** The acaricides used in this study.

Acaricides	Manufacturer
Cyetpyrafen 98% a.i.	Shenyang NovPharm Technology Co., Ltd. (Liaoning, China)
Cyetpyrafen 30% SC	
Cyenopyrafen 95% a.i.	Nissan Chemical Ind., Ltd. (Shanghai, China)
Cyenopyrafen 30% SC	
Bifenazate 98% a.i.	Qingdao Runnong Chemical Co., Ltd. (Qingdao, China)
Bifenazate 43% SC	Qingdao Zhongda Agritech Co., Ltd. (Qingdao, China)
Abamectin 96% a.i.	Shandong Jingbo Agrochemicals Technology Co., Ltd. (Binzhou, China)
Abamectin 3% ME	Shaanxi Sunger Road Bio-science Co., Ltd. (Xi’an, China)
Azocyclotin 95% a.i	Zhaoyuan Sanlian Chemical Co., Ltd. (Yantai, China)
Azocyclotin 20% SC	Shandong Qixia Tongda Chemical Co., Ltd. (Yantai, China)
Spirodiclofen 98% a.i.	Hailir Pesticides and Chemicals Group Co., Ltd. (Qingdao, China)
Spirodiclofen 240 g/L SC	Hailir Pesticides and Chemicals Group Co., Ltd. (Qingdao, China)
Etoxazole 96% a.i.	Hunan Haohua Chemical Co., Ltd. (Zhuzhou, China)
Etoxazole 30% SC	Shaanxi Sunger Road Bio-science Co., Ltd. (Xi’an, China)
Cyflumetofen 98% a.i.	Shandong Sino-Agri United Biotechnology Co., Ltd. (Jinan, China)

a.i. = active ingredient; SC = suspension concentrate; and ME = microemulsion.

## Data Availability

The data are available from the corresponding author upon reasonable request.
